# Genetic analysis and molecular characterization of Chinese sesame (*Sesamum indicum* L.) cultivars using Insertion-Deletion (InDel) and Simple Sequence Repeat (SSR) markers

**DOI:** 10.1186/1471-2156-15-35

**Published:** 2014-03-19

**Authors:** Kun Wu, Minmin Yang, Hongyan Liu, Ye Tao, Ju Mei, Yingzhong Zhao

**Affiliations:** 1 Key Laboratory of Biology and Genetic Improvement of Oil Crops, Ministry of Agriculture, Sesame Genetic Improvement Laboratory, Oil Crops Research Institute of the Chinese Academy of Agricultural Sciences (OCRI-CAAS), Wuhan, Hubei 430062, China; 2 Shanghai Major Biological Medicine Technology Co., Ltd, Shanghai 201203, China

**Keywords:** Sesame, InDel, Microsatellites, Genetic diversity, Population structure, Allele distribution

## Abstract

**Background:**

Sesame is an important and ancient oil crop in tropical and subtropical areas. China is one of the most important sesame producing countries with many germplasm accessions and excellent cultivars. Domestication and modern plant breeding have presumably narrowed the genetic basis of cultivated sesame. Several modern sesame cultivars were bred with a limited number of landrace cultivars in their pedigree. The genetic variation was subsequently reduced by genetic drift and selection. Characterization of genetic diversity of these cultivars by molecular markers is of great value to assist parental line selection and breeding strategy design.

**Results:**

Three hundred and forty nine simple sequence repeat (SSR) and 79 insertion-deletion (InDel) markers were developed from cDNA library and reduced-representation sequencing of a sesame cultivar Zhongzhi 14, respectively. Combined with previously published SSR markers, 88 polymorphic markers were used to assess the genetic diversity, phylogenetic relationships, population structure, and allele distribution among 130 Chinese sesame accessions including 82 cultivars, 44 landraces and 4 wild germplasm accessions. A total of 325 alleles were detected, with the average gene diversity of 0.432. Model-based structure analysis revealed the presence of five subgroups belonging to two main groups, which were consistent with the results from principal coordinate analysis (PCA), phylogenetic clustering and analysis of molecular variance (AMOVA). Several missing or unique alleles were identified from particular types, subgroups or families, even though they share one or both parental/progenitor lines.

**Conclusions:**

This report presented a by far most comprehensive characterization of the molecular and genetic diversity of sesame cultivars in China. InDels are more polymorphic than SSRs, but their ability for deciphering genetic diversity compared to the later. Improved sesame cultivars have narrower genetic basis than landraces, reflecting the effect of genetic drift or selection during breeding processes. Comparative analysis of allele distribution revealed genetic divergence between improved cultivars and landraces, as well as between cultivars released in different years. These results will be useful for assessing cultivars and for marker-assisted breeding in sesame.

## Background

Sesame (*Sesamum indicum* L.) (2n = 26) is an important and ancient oilseed crop in tropical and subtropical regions of Asia, Africa and South America [1]. It is a diploid species belonging to the *Sesamum* genus of Pedaliaceae family with an estimated genome size of ~369 Mb [2]. Sesame seeds are considered to have the highest oil contents among major oilseed crops including also peanut, soybean and rapeseed [3]. It is also rich in proteins, vitamins and antioxidants such as sesamin and sesamolin [4,5]. China is one of the most important sesame producing countries that contributes over 20% and consumes ~30% of the world’s production, with the highest yield level around the world (2001 to 2010, UN Food and Agriculture Organization Data).

There are currently 4251 accessions in the Chinese sesame germplasm collection. More than 80 cultivars were released in the period between 1950 and 2012 [6,7]. Despite of the number of commercial cultivars, a main hindrance in sesame production is the lack of cultivars with high-yield stability and adaptability. Domestication and modern plant breeding have presumably narrowed the genetic basis of cultivated sesame, as has been in wheat, maize and other field crops [6,8,9]. These modern sesame cultivars were bred with a limited number of landrace cultivars in their pedigree. For example, more than 12 important improved cultivars including the well know Yuzhi 4, Wanzhi 2, Ezhi 6, Zhongzhi 11 and Zhongzhi 12 were developed from a common parent of Yiyangbai, directly or indirectly. Assessment of genetic variation among these modern and landrace sesame cultivars can provide breeders with insight into the need to introgress more elite germplasm into their programs to broaden genetic variation.

It is necessary to take reliable identification of these sesame cultivars through DNA fingerprinting by molecular markers, which has been widely used for checking the identity and purity of cultivars and for assessing their genetic variability in different crops [8-13]. In sesame, the genetic diversity has been detected by universal markers such as amplified fragment length polymorphism (AFLP) [14,15], sequence-related amplified polymorphisms (SRAP) [6,7,16], random amplified polymorphic DNA (RAPD) [17-19] and inter-simple sequence repeat (ISSR) [20,21]. Applications of sequence-specific markers such as genomic simple sequence repeats (Genomic-SSR) [22-24] and expressed sequence tag-SSR (EST-SSR) [25,26] were also documented. Since most of the aforementioned studies used only limited number of improved cultivars or markers, a more comprehensive analysis of common sesame cultivars in a nationwide level is required to reach a definitive understanding of their genetic variation.

SSRs are short (1-8 bp) repeat motifs usually associated with high frequency of length polymorphism. With the advantages of simplicity, effectiveness, abundance, reproducibility, codominant inheritance and extensive genomic coverage, SSRs have been applied to disclose genetic diversity and relationship in a number of crop species [27-32]. Few polymorphic SSR markers have been identified in sesame [22-26,33]. In addition, Insertion-Deletion (InDel) markers, which arise from insertion of transposable elements, slippage in simple sequence replication or unequal crossover events, also share these advantages for SSRs [34]. InDels have also been widely applied for genotyping, genetic diversity analysis, QTLs mapping, map-based cloning, and even marker-assisted selections in Arabidopsis, rice, wheat, turnip, sunflower, citrus, and Atlantic salmon [35-43].

In this study, we developed and characterized 349 EST-SSR markers from a cDNA library [44], and 76 InDel markers from a reduced-representation gDNA library of the same commercial sesame cultivar Zhongzhi 14. We applied these newly developed markers with 600 published EST-SSR or Genomic-SSR markers to 82 improved cultivars or inbred lines, which collectively represent virtually all the available Chinese improved sesame cultivars, and made comparison with the results from assessing 48 important landraces or wild germplasm accessions.

## Results

### Development and characterization of sesame SSRs and InDels

For those 1,949 non-redundant SSRs identified from unigenes of ‘Zhongzhi 14′ [44], 349 primer pairs named as SBM series were successfully designed and synthesized for genetic diversity analysis in sesame (Additional file 1: Table S1). Superadded previously published sesame SSRs, a total of 815 EST-SSRs and 134 genomic-SSRs were surveyed on the genomic DNA of ‘Zhongzhi 14′ and ‘Miaoqianzhima’. As a result, 82.52% EST-SSR and 79.85% genomic-SSR primer pairs generated reproducible and clear amplicons in two reference templates. Among these markers, 39 EST-SSRs (5.17%) and 13 genomic-SSRs (12.15%) detected polymorphisms (Table 1).

**Table 1:** Types of markers surveyed and the polymorphism detection rates between ‘Zhongzhi 14′ and ’Miaoqianzhima’

**Type**	**Series code**	**Number**	**Number (%) of markers with**		**Source**
			**Clear bands**	**Detecting polymorphism**^**a**^	
Genomic-SSR	GBssr, Saesam	23	22 (95.7)	4 (18.2)	Dixit et al. [22]; Cho et al. [24]
	No. (named ‘GSSR’)	111	85 (76.6)	9 (10.6)	Spandana et al. [45]
EST-SSR	ZHY	25	24 (96.0)	2 (8.3)	Wei et al. [33]
	HS	342	316 (93.0)	14 (4.4)	Zhang et al. [25]; Yue et al. [46]
	ZM	99	99 (100.0)	12 (12.1)	Wei et al.[59]; Wang et al. [26]
	SBM	349	315 (90.3)	11 (3.5)	Authors’ laboratory
InDel	SBI	79	75 (94.9)	36 (48.0)	Authors’ laboratory

^a^% Number of markers detecting polymorphism VS number of markers producing clear bands.

Ninety-seven InDels were identified through comparative Restriction-site Associated DNA (RAD) sequencing of the genomes of ‘Zhongzhi 14′ and ‘Miaoqianzhima’, with the GenBank accession numbers KG777470-KG777548. And 79 primer pairs were successfully designed and synthesized for genetic diversity analysis (Additional file 2: Table S2). As a result, 75 primer pairs generated single and clear bands as expected. And 36 InDels detected repeatable polymorphisms between two references (Table 1).

**Figure 1:**
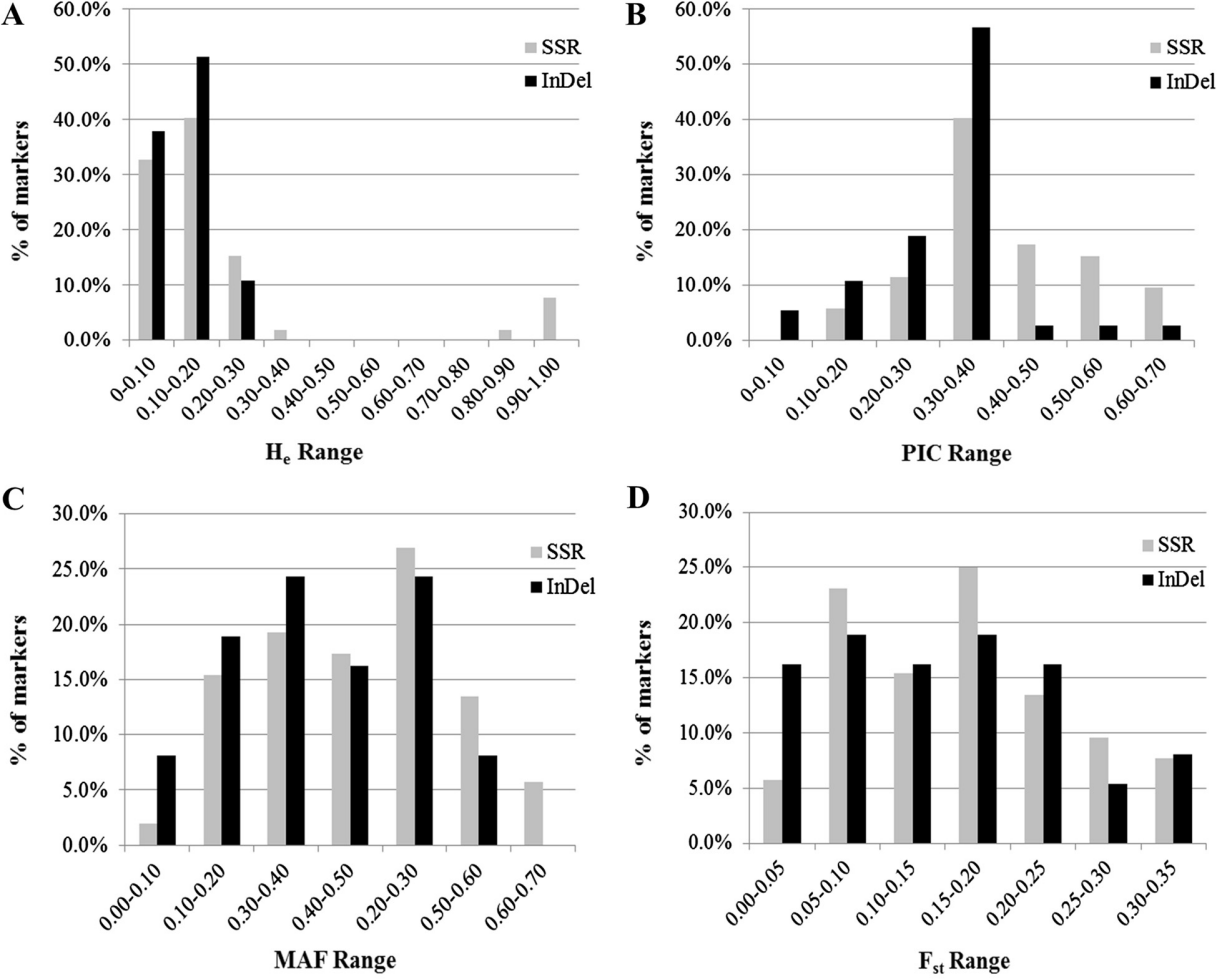
**Comparison the distribution of observed heterozygosity (H**_**e**_**) (A), polymorphic information content (PIC) (B), minor allele frequency (MAF) (C) and F-statistics (F**_**st**_**) (D) between SSR and InDel markers.**

Then, 88 primer pairs, including 39 EST-SSRs, 13 genomic-SSRs and 36 InDels, that amplified reproducible and polymorphic bands were used to genotype 130 sesame cultivars, landraces or wild germplasm. A total of 223 and 102 alleles were detected using SSR and InDel markers, respectively. Allele number per locus for SSR and InDel markers ranged from 2 to 9 and from 2 to 6 (with average number of 4.29 and 2.76), H_e_ average was 20.7% and 12.0%, gene diversity average was 0.47 and 0.39, PIC average was 0.40 and 0.32, average minor allele frequency (MAF) was 35.58% and 28.78%, average *F*_*st*_ was 0.16 and 0.15, respectively. And the average alleles number per locus, gene diversity and PIC of SSR markers were significant higher than InDel markers (*P* < 0.01). The distribution of H_e_, MAF and *F*_*st*_ among the whole population confirmed that InDel markers are less polymorphic than SSR markers but showed similar differentiation between sesame accessions (Figure 1). The observed H_e_ was obviously lower for InDel than SSR markers. The MAF and *F*_*st*_ values were similar between InDel and SSR markers. So these InDel and SSR markers showed comparable ability in deciphering genetic diversity of sesame in this study.

### Genetic diversity

**Figure 2:**
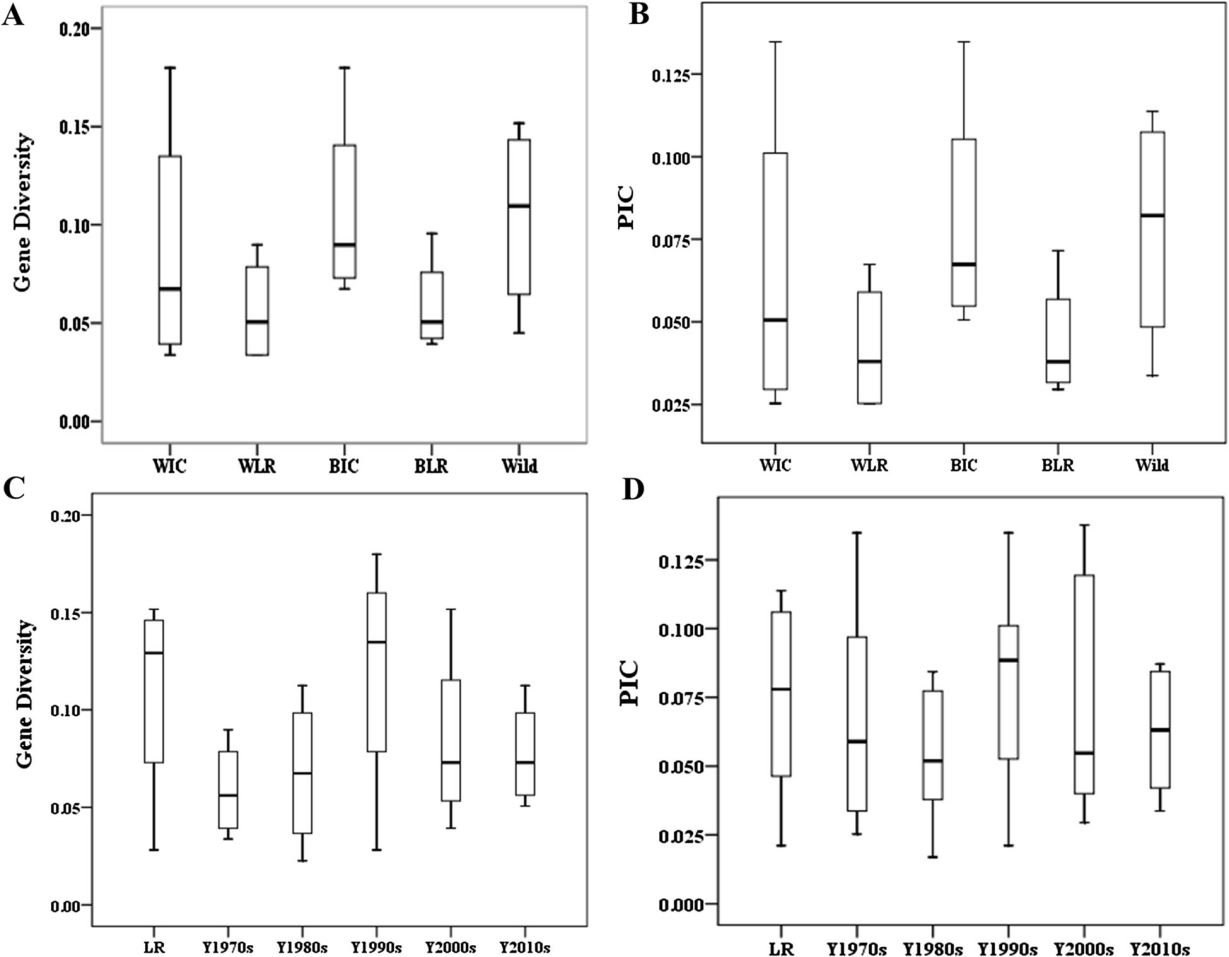
***Box *and *Whisker box *of summary statistics for 325 SSR or InDel loci in five different subsets by types (A, B) or releasing period of cultivars (C, D). A and C gene diversity; B and D polymorphic information content (PIC).** WIC[L], White seeded Improved cultivars or Inbred lines; WLR, White seeded Landraces; BIC, Black seeded Improved cultivars; BLR, Black seeded Landraces; LR refer to white or black seeded Landraces and four wild accessions; Y1970s, Y1980s, Y1990s, Y2000s and Y2010s refer to improved cultivars released in or prior to the 1970s, in the 1980s, 1990s, 2000s and 2010s, respectively.

Genotyping of 130 individuals including white seeded improved cultivars or inbred lines [WIC(L)], white seeded landraces (WLR), black seeded improved cultivars (BIC), black seeded landraces (BLR) and wild germplasm accessions revealed a total of 325 alleles. The average allele number per locus for the five different subsets varied from 2.3034 to 2.9213, with the highest number in wild germplasm accessions. Four wild germplasm accessions showed higher MAF, gene diversity, heterozygous and PIC than the rest four subsets. Seventy WIC(L) accessions had the significantly lowest MAF, gene diversity and PIC values (*P* < 0.01) (Table 2). Compared to the WLR or BLR, respectively, WIC(L) and BIC had significantly higher level of gene diversity and PIC (Figure 2A, B). Furthermore, these improved cultivars (including both white and black seeded) were also compared for genetic diversity among subsets by releasing period (Table 2). Compared to landraces, the five subsets including Y1970s, Y1980s, Y1990s, Y2000s and Y2010s cultivars had lower MAF, gene diversity and PIC values. Landraces and Y1990s cultivars had similarly higher heterozygosity level than other three subsets. The MAF, gene diversity and PIC of Y2010s cultivars were significantly lower than those of all other subsets (*P* < 0.05) (Table 2). For gene diversity, Y1990s cultivars had the largest variation, followed by Y2000s and Y1980s (Figure 2C). The variations of PIC within Y1970s, Y1990s and Y2000s were similarly higher than those in Y1970s and Y2010s (Figure 2D).

**Table 2:** Statistical summary of the genetic diversity of five different sesame subsets

**Subsets**	**Accession Number**	**MAF**	**Genotype number**	**Allele number**	**Gene Diversity**	**Heterozygosity**	**PIC**
WIC[L]	70	0.2714**	3.5169	2.4157	0.3635**	0.1535	0.3047**
WLR	31	0.3337	3.6067	2.5843	0.4306	0.1769	0.3588
BIC	12	0.3172	2.8315*	2.3034**	0.4066	0.1464	0.3346
BLR	13	0.3124	3.0674	2.4719	0.4098	0.1858	0.3425
Wild	4	0.4232	2.6854**	2.9213	0.5221	0.3801	0.4564
Y1970s	8	0.2667	2.3820*	2.0674*	0.3524	0.1701	0.2905
Y1980s	10	0.2891	2.6180	2.2135	0.3788	0.1236**	0.3151
Y1990s	10	0.2612	2.8090	2.2247	0.3502	0.1989	0.2925
Y2000s	36	0.2773	3.4607	2.4045	0.3724	0.1450*	0.3135
Y2010s	12	0.2525*	2.6180	2.1348	0.3358*	0.1490	0.2781*
LR	48	0.3742	4.9663	3.6067	0.4791	0.1911	0.4056
P1	28	0.2424	3.0337	2.2921	0.3313	0.1409	0.2795
P2	23	0.1975	2.6180**	2.0337**	0.2596**	0.1251*	0.2152**
P3	14	0.2582	2.7978	2.2022	0.3429	0.2064	0.2850
P4	13	0.2736	2.9101	2.3371	0.3628	0.1495	0.3049
P5	4	0.4232**	2.6854*	2.9213	0.5221	0.3801	0.4564
Overall		0.3265	5.1798	3.6517	0.4323	0.1686	0.3650

Mean values are represented in the table, *P ≤ 0.05 and **P ≤ 0.01 were assessed by Z-test.

Subsets of WIC[L], WLR, BIC, BLR and Wild includes 130 accessions; Subsets of Y1970s, Y1980s, Y1990s, Y2000s, Y2010s and LR (WLR, BLR or wild accessions) includes 124 accessions except 6 white seeded inbred lines; Subsets of subgroup P1, P2, P3, P4 and P5 includes 82 accessions except 48 accessions that classified into a mixed subgroup.

### Population structure and genetic clustering

**Figure 3:**
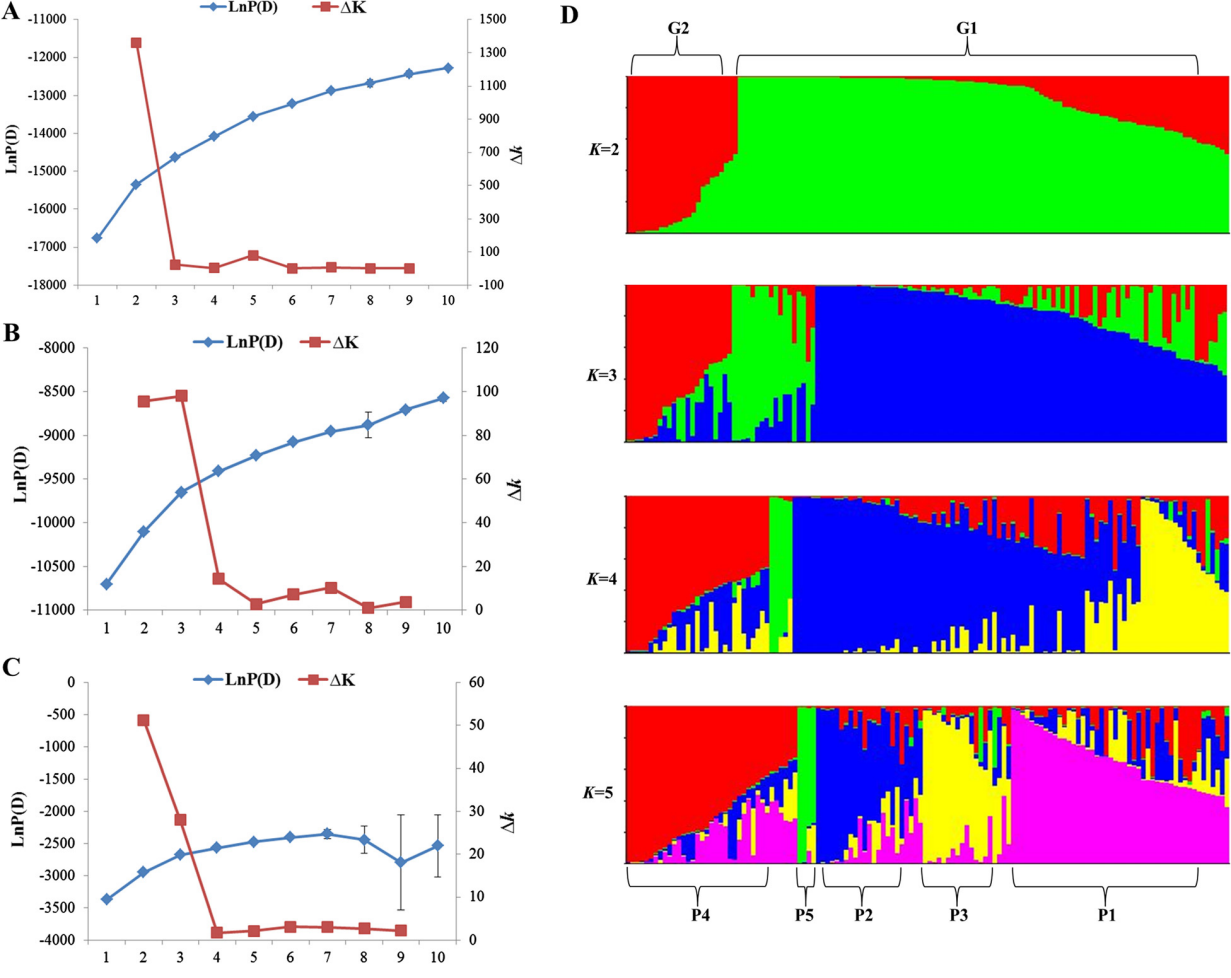
**Analysis of the population structure based on 88 SSR or InDel markers. A** Estimated LnP(D) and ∆*k* of total 130 sesame lines over five runs for each *k* value. **B** Estimated LnP(D) and ∆*k* of 98 lines in G1 over five runs for each *k* value. **C** Estimated LnP(D) and ∆*k* of 21 lines in G2 over five runs for each *k* value. **D** Estimated population structure in 130 sesame lines assessed by STRUCTURE. Each individual is represented by a thin vertical bar, partitioned into up to *k* colored segments.

To examine the relatedness among these 130 lines, the genotypic data for 52 SSRs and 36 InDels were analyzed using a model-based approach implemented in STRUCTURE. Fifty datasets were obtained by setting the number of possible clusters (*k*) from 1 to 10 with five replications each. The LnP(D) for each given *k* increased with the increase of *k* and the most significant change was observed when *k* increased from 1 to 2. In addition, a sharp peak of the second-order likelihood, ∆*k*, appeared at *k* = 2 (Figure 3A). Accordingly, the total panel could be divided into two main groups, designated as G1 and G2, respectively (Figure 3D, Additional file 3: Table S3). The G1 group contained 98 lines, most of which are white seeded. The G2 group contained 21 lines, mostly black seeded (Additional file 3: Table S3). The remaining 11 lines each had a membership probability lower than 0.60 in any given group and were thus classified into a mixed group (named Gmix). The main groups were further subdivided into P1, P2, P3 and P4, P5 subpopulations, respectively, as suggested by the STRUCTURE analysis (Figure 3). The P1 subgroup included 21 WIC(L)s and 7 WLRs (53.6% from Hubei Province). The P2 subgroup included 21 WIC(L)s, one BIC and one WLR (56.5% from Henan Province). The P3 subgroup included 5 WICs, 8 WLRs, and one BIC. The P4 subgroup contained 5 BICs (all from Jiangxi Province), 7 BLRs (such as Wuninghei, most from south China or Asia) and one WLR (C-50, from India). The P5 subgroup included only four wild germplasm accessions from India or Africa. The remaining 48 lines were classified into a mixed subgroup (named Pmix) as they had membership probabilities lower than 0.60 in any given subgroup (Additional file 3: Table S3).

**Figure 4:**
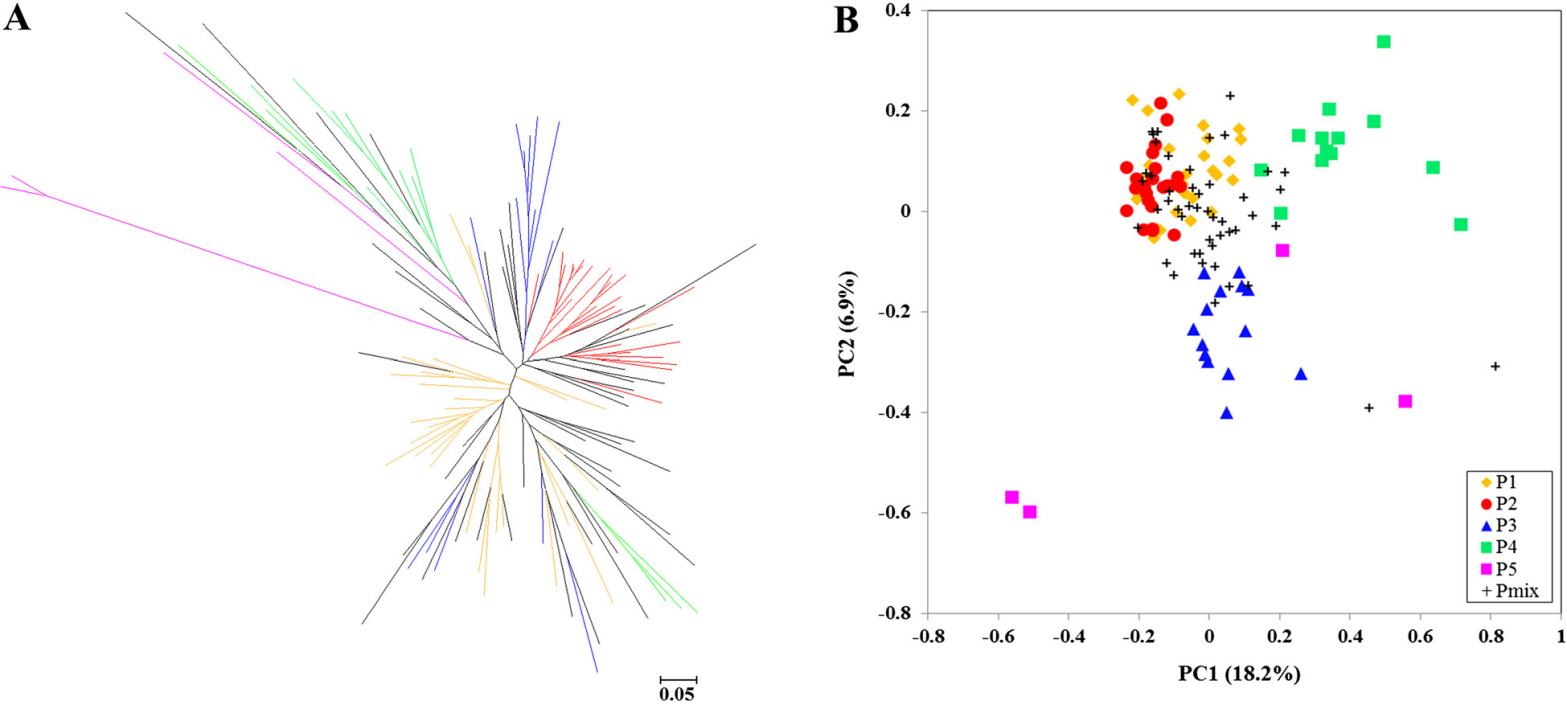
**Representation of genetic structure of 130 sesame lines based on Neighbor-joining phylogenetic tree (NJ-tree) (A) and Principal component analysis (PCA) (B). P1, P2, P3, P4, P5 and Pmix are subgroups identified by STRUCTURE assigned with the maximum membership probability.** For NJ-tree and PCA plot, the different colored lines or plots represent the different subgroups inferred by STRUCTURE analysis. P1 yellow, P2 red, P3 blue, P4 green, P5 pink, Pmix black.

Moreover, we also constructed a neighbor-joining tree and conducted PCA to examine genetic population structure and genetic clustering of these sesame accessions. The NJ phylogenetic tree based on Nei’s genetic distances (1972) displayed a similar clustering pattern of relationship to that of STRUCTURE (Figure 4A). The tree had five clear branches with the “mixed” lines (Pmix, in black) distributed in each branch. PCA based on Nei’s genetic distances showed a similar, five-cluster distribution pattern, with the mixed subgroup being in the middle of these five defined subgroups (Figure 4B). The top two principal components clearly separated these subgroups, but only partially distinguished P1 and P2. It appeared that P3, P4 and P5 were relatively distant from P1 and P2, which were close to each other. P3 and P4 were distant from each other. More interestingly, Wild 1 and Wild 2 from P5 were genetically far away from the rest four subgroups, while other two wild germplasm accessions were comparatively closer to P4 and P3.

### Population differentiation and diversity

AMOVA was performed and *F*_*st*_ was calculated to investigate population differentiation and diversity. AMOVA results indicated that only 10.23% (*P* < 0.001) of the total molecular variation was partitioned among groups, 20.23% (*P* < 0.001) was attributed to differentiation among subgroups and 69.54% (*P* < 0.001) within subgroups. Pairwise *F*_*st*_ of the two inferred groups was 0.19 (*P* < 0.001), suggesting that G1 is largely divergent from G2. The levels of differentiation between subgroups were varied, with *F*_*st*_ ranging from 0.19 (P1 and P2, *P* < 0.001) to 0.41 (P2 and P5, *P* < 0.001) (Table 3). A similar pattern of differentiation among subgroups was also observed using Nei’s minimum distance, which ranged from 0.12 to 0.47 with the correlation coefficient to *F*_*st*_ being 0.704 (*P* < 0.05) (Table 3).

**Table 3:** **Genetic distance, as measured by Nei’s (1973) minimum distance (top diagonal) and pairwise *F***_***st ***_**comparisons (bottom diagonal) among inferred sesame subgroups**

**Group**	**Subgroup**	**G1**			**G2**	
		P1	P2	P3	P4	P5
G1	P1		0.12	0.19	0.28	0.43
	P2	0.19**		0.21	0.32	0.46
	P3	0.24**	0.29**		0.30	0.40
G2	P4	0.30**	0.37**	0.30**		0.47
	P5	0.33**	0.41**	0.28**	0.29**	

**Significant at *P* < 0.01 after 1,000 permutations.

The genetic diversity in inferred subgroups was also assessed and compared using MAF, gene diversity, heterozygosity and PIC (Table 2). Compared to the entire panel, P2 had significantly lower gene diversity, allele number per locus, heterozygosity and PIC (*P* < 0.05 or *P* < 0.01). P5 had the highest level of MAF among all subgroups, followed by P4, P3, P1 and P2. P3 exhibited a similar level of MAF, gene diversity and PIC to P1 and P4, but higher level of heterozygosity (*P* < 0.01).

### Allele frequencies and alleles distribution in different sesame cultivars in China

**Figure 5:**
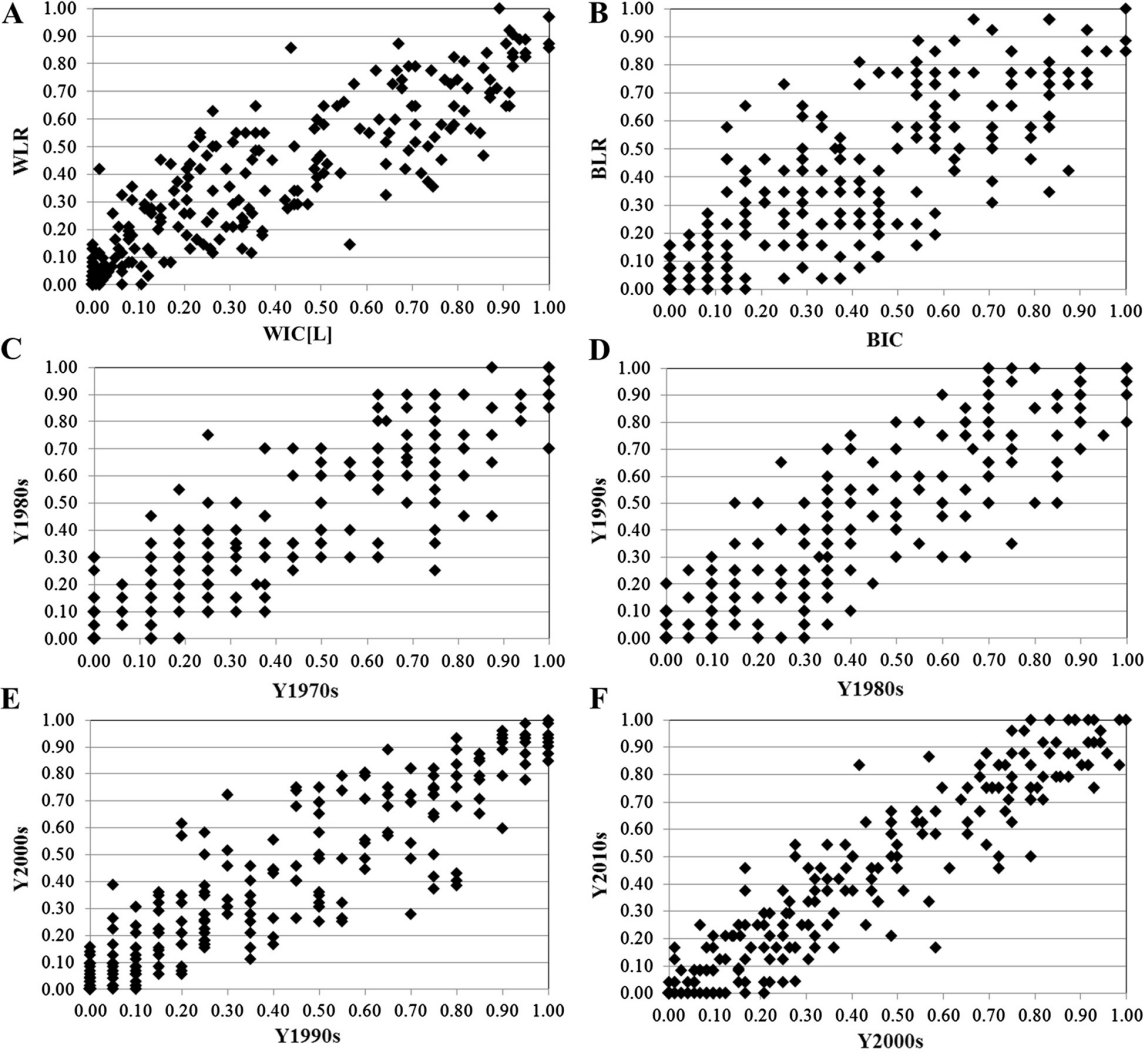
***X*-*Y *plots for allele frequencies in pairwise comparisons of sesame accessions. A** WIC(L) versus WLR, **B** BIC versus BLR, **C** Y1980s versus Y1970s, **D** Y1990s versus Y1980s, **E** Y2000s versus Y1990s, **F** Y2010s versus Y2000s, respectively.

To more deeply dissect the genetic differentiation among different set of sesame cultivars in China, comparative analysis of allele frequencies was performed (Additional file 4: Table S4). Of the 325 alleles, allele frequencies difference larger than 10% (*P* > 0.01) were observed for 117 (36.0%) alleles in the WIC(L) versus WLR subgroup (Figure 5A), and 133 (40.9%) alleles in the BIC versus BLR subgroup (Figure 5B). In comparison with the WLR subgroup, there were 22 missing alleles and 7 unique alleles identified in WIC(L). And 21 missing alleles and 6 unique alleles were identified in BIC subgroup compared to BLR. (Additional file 4: Table S4).

We also compared the allele frequencies of sesame cultivars that were released in different timelines to reveal their genetic difference. In the Y1980s versus Y1970s and Y1990s versus Y1980s comparisons, respectively, 125 (38.5%) and 134 (41.2%) alleles showed an allele frequency difference larger than 10% (*P* < 0.01) (Figure 5C, D). Only 88 (27.1%) and 68 (20.9%) alleles had an allele frequency larger than 10% in the comparisons of Y2000s versus Y1990s and Y2010s versus Y2000s, respectively (Figure 5E, F). Compared to the Y2000s subset, only 1 unique allele but 25 missing alleles were identified in the Y2010s subset (Additional file 4: Table S4). These results indicate distinct genetic differences among the four pairwise comparisons, with the strongest differentiation between Y1980s and Y1970s lines or Y1990s and Y1980s, the second between Y2000s and Y1990s, and the least between Y2010s and Y2000s (Figure 5C to F).

Moreover, we also compared the distribution of 325 alleles in four important Chinese sesame cultivar families with four different parental/progenitor lines (Table 4). In family I with the common parental/progenitor of Yiyangbai, two cultivars were from subgroup P1, three from P2, and 5 from Pmix. They shared 27 common alleles, such as SBM073.5, HS050.2, ZM0740.1 and SBI009.3 (Table 4). Cultivars from the family II with Yuzhi No.4 as the common donor shared 22 alleles, most of which were from P2 subgroup, except for Wanzhi No.1, Zhuzhi No.11 and other four lines (Table 4). The family III of Zhongzhi No.1 included 4 cultivars from P1, 3 from Pmix and one from P1, with 21 shared alleles (Table 4). While the black seed-type family IV of Wuninghei had 4 cultivars with 19 shared alleles. On the whole, three EST-SSRs alleles and three genomic InDels alleles were shared in four families, including SBM073.8, SBM768.6, HS050.2, SBI014.1, SBI017.2 and SBI019.2. And six alleles including SBM750.3, SBM1111.1, HS137.4, Y1972.1, ZHY01.3 and SBI060.1 were found to be specially shared in familyI. Four alleles including HS225.1, ZM1179.2, SBI023.2 and SBI034.1 were specially shared in family II. Other four alleles of GSSR007.2, SBI036.2, SBI050.1 and SBI071.2 were specially shared in family III. Eight alleles specially shared in family IV were also be identified, including SBM768.5, ZM1413.2, ZM1488.1, SBI005.1, SBI007.4, SBI025.1, SBI027.2 and SBI051.3 (Table 4). These alleles identified above with different allelic frequency, even miss, unique or family special, can be combined and used for characterization of sesame cultivars and for sesame molecular breeding.

**Table 4:** Comparison of cultivars from four different families using 89 molecular markers

**Family**^**a**^	**Cultivar**	**Time**^**b**^	**Parentage**	**Group**	**Shared alleles**^**d**^
**I**	Yiyangbai^c^	1970s	Selection from variety of “Zhongxiang Huangzhima”	P1	SBM073.5, SBM073.8, SBM750.3, SBM768.6, SBM1111.1, SBM1120.2, HS050.2, HS137.4, HS142.3, HS176.3, Y1972.1, ZHY01.3, ZHY023.4, ZM0740.1, ZM0961.4, GB182.3, SBI009.3, SBI012.2, SBI014.1, SBI017.2, SBI019.2, SBI030.1, SBI041.4, SBI043.2, SBI054.2, SBI060.1, SBI064.1
	Ningzhi No.1	1980s	Selection from variety of “Yiyangbai”	Pmix	
	Yuzhi No.4	1980s	Yiyangbai × Zhuzhi No. 1	P2	
	Yuzhi No.7	1990s	Zhongzhi No.7 × Yiyangbai	Pmix	
	Yuzhi 18	2000s	(Variety of Yiyangbai × Yuzhi No. 11)F_3_ × Zhenzhi 958	P2	
	Ezhi No. 6	2000s	Ezhi No. 1 × Yiyangbai	Pmix	
	Zhongzhi 12	2000s	CLSU-9 (Philippine) × Yiyangbai	P1	
	Zhongzhi 18	2010s	(Yiyangbai × Ezhi No. 1)F_2_ × Zhongzhi 11	Pmix	
	Zhongzhi 21	2010s	[(Yiyangbai × Zhushanbai) F_4_] × Fufengzhima	Pmix	
	Zhongzhiza No.2	2010s	95 ms-2 (male sterile) × Zhongzhi 12	P2	
**II**	Yuzhi No.4^c^	1980s	Yiyangbai × Zhuzhi No. 1	P2	SBM064.3, SBM073.5, SBM073.8, SBM768.6, SBM1120.2, HS050.2, HS123.3, HS142.3, HS176.3, HS225.1, ZHY023.4, ZM030.2, ZM0740.1, ZM0961.4, ZM1179.2, SBI014.1, SBI017.2, SBI019.2, SBI023.2, SBI034.1, SBI043.2, SBI064.1
	Luozhi 12	2000s	Zhen 89H142 × Yuzhi No. 4	Pmix	
	Luozhi No. 15	2000s	Variety of Yuzhi No. 4	P2	
	Wanzhi No. 1	2000s	0176A (male sterile) × Yuzhi No. 4	P1	
	Wanzhi No. 2	2000s	Fuyang Xiaozibai × Yuzhi No. 4	P2	
	Zhongzhi 11	2000s	Space mutant of Yuzhi No. 4	P2	
	Zhongzhi 13	2000s	Space mutant of Yuzhi No. 4	P2	
	Zhongzhi 14	2000s	85-411 × Yuzhi No. 4	Pmix	
	Zhongzhi 15	2000s	Yuzhi No. 4 × Suxianzhima	Pmix	
	Zhuzhi No. 11	2000s	Zhu 81043 × Zhu 7801 (variety of Yuzhi No. 4)	P1	
	Zhuzhi No. 14	2000s	Zhu 86036 × Zhu 7801 (variety of Yuzhi No. 4)	P2	
	Zhuzhi No. 18	2000s	Zhu 893 × Zhu 7801 (variety of Yuzhi No. 4)	P2	
	Ezhi No. 7	2010s	Ezhi No. 3 × Yuzhi No. 4	Pmix	
**III**	Zhongzhi No.1^c^	1970s	Selection from “Enshi Baizhima”	Pmix	SBM064.3, SBM073.8, SBM768.6, HS050.2, HS176.3, ZM030.2, ZM0740.1, GB182.3, GSSR007.2, GSSR090.4, SBI009.3, SBI014.1, SBI017.2, SBI019.2, SBI030.1, SBI036.2, SBI043.2, SBI050.1, SBI054.2, SBI064.1, SBI071.2
	Zhongzhi No.7	1970s	Xiangyang Xiniujiao × Zhonghzi No.1	P1	
	Zhongzhi No.8	1980s	Zhongzhi No. 7 × Jiangling Yongguangxingzhima	Pmix	
	Zhongzhi No.9	1990s	Xinjiang Heizhima × Zhongzhi No. 7	Pmix	
	Zhongzhi No.10	1990s	{Zhongzhi No. 5 × [(Zihuayeersan × Zhongzhi No. 1) × Suiping Xiaozihuang]} × (Zhongzhi No. 5 × Zhecheng Tiegucha)	P1	
	Zhongzhi 17	2010s	Zhu86-207 × Zhongzhi No. 10	P2	
	Zhongzhi 22	2010s	Zhongzhi No. 10 × Ezhi No. 1	P1	
	Zhongzhi 23	2010s	(Zhongzhi No. 10 × Zhu 04) × Zhenzhi 98 N09	P1	
**IV**	Wuninghei^c^	1980s	An important landrace	P4	SBM064.3, SBM073.8, SBM768.5, SBM768.6, HS050.2, HS123.3, ZM1413.2, ZM1488.1, GSSR090.4, SBI005.1, SBI007.4, SBI012.2, SBI014.1, SBI017.2, SBI019.2, SBI025.1, SBI027.2, SBI030.1, SBI051.3
	Ganzhi No. 6	2000s	Yujiangheizhima × Wuninghei	Pmix	
	Ganzhi No. 9	2000s	Co^60^ radiation mutant of “Wuninghei”	P4	
	Jiheizhi No. 1	2000s	Jizhi No. 1 × Wuninghei	P3	
	Zhuzhi No. 10	2000s	7801H (variety of Yuzhi No. 4) × Wuninghei	Pmix	

^a^Four different families with one common parent or progenitor. ^b^release or application time of these cultivars. ^c^the corresponding common parents or progenitors of each family. ^d^the shared genetic alleles in one family.

## Discussion

### Development and utilization of sesame SSR and InDel markers for sesame genetic diversity analysis

In this study, we developed 315 EST-SSR markers from 1,688 unigenes from sequencing a cDNA library of Zhongzhi 14. Combined with 466 earlier EST-SSR and 134 earlier genomic-SSR markers in sesame, only 5.17% EST-SSRs and 12.15% genomic-SSRs (gSSRs) showed polymorphism between ‘Zhongzhi 14′ and ‘Miaoqianzhima’, which were two parents of an important RIL population for other works. Such polymorphism rate of EST-SSRs is lower than that in an intraspecific cross (7.5% or 6.52%) [25,33], but higher than that of 36 sesame accessions (4.01%) [26]. Polymorphism rate of gSSRs in this study is lower than reported in two earlier studies [22,45], which were 20% and 26.3% respectively. The relative low level of SSR polymorphism between ‘Zhongzhi 14′ and ‘Miaoqianzhima’ is obviously inconsistent with their obviously morphological variations, which might be interpreted by InDel, SNP (single nucleotide polymorphism), methylation or other genomic variation. And more polymorphic SSR markers might be identified by using more genomic sequence and more DNA template of sesame accessions.

A total of 75 genomic InDel markers were also developed, making use of RAD sequencing of ‘Zhongzhi 14′ and ‘Miaoqianzhima’. The InDel markers showed much higher ability to discern genetic diversity, as the rate of polymorphism is as high as 48.0%. In the collection of cultivars, landraces even wild germplasm with different chromosome numbers, most InDel markers yielded single PCR fragments and showed polymorphisms. Such high efficiency of InDel markers was also reported in *Brassica rapa*, *Arabidopsis*, *Helianthus annuus* and *Citrus*[35,36,38,39,41]. Furthermore, the average allele number per locus, H_e_, gene diversity and PIC of SSR markers were significant higher than those of InDel markers in the whole panel, as opposed to MAF and *F*_*st*_ values, which were similar between InDel and SSR markers. The distribution of H_e_, MAF and *F*_*st*_ further confirmed that InDel markers showed similar differentiation between sesame accessions with more polymorphic than SSR markers. Similar pattern was also reported in cultivated citrus [41]. Therefore, this set of novel PCR-based SSR and InDel markers will be valuable for genetic studies and breeding in sesame. In addition, most of these polymorphic SSR and InDel markers showed normal segregation in a RIL population (data not shown), based on which a project toward high density genetic mapping employing these SSRs, InDels plus some SNP markers is now underway in our lab.

### Genetic diversity and population structure in sesame panel

A thorough understanding of genetic diversity, population structure and familiar relatedness in a given panel is very important for successful association studies. For this purpose, a large number of DNA markers that are genome-wide distributed, reproducible, cost-effective, selectively neutral and highly polymorphic are necessary. SSRs and InDels are two nice choices of this kind. In this study, 88 polymorphic markers including EST-SSRs, genomic-SSRs and InDels randomly distributed in *Sesamum indicum* L. genome were selected to evaluated 130 sesame cultivars, landraces or wild germplasm. A total of 325 alleles, with an average of 3.69 alleles per locus, were detected in this sesame panel. The number of polymorphic markers used in this study is higher than in most earlier reports, but the number of allele per locus is lower than that detected in 150 [24], 453 [7], 545 [46], 216 [47] sesame accession and 67 sesame cultivars in China [6]. The difference of allelic richness between our panel and other germplasm collections may be caused by the differences of materials analyzed, but the use of only site-specific SSR and InDel markers may also account for this.

More importantly, a larger number of loci (in particular, the use of dinucleotide repeat SSRs than tri- or higher) will lead to a higher number of alleles and thus a higher apparent level of genetic diversity [48]. The average PIC value and gene diversity across all lines in this panel were 0.365 and 0.432, respectively. They were much higher than some reported values [14,16,47,49,50], but lower than those of Yue et al. (2012) and Cho et al. (2011) [24,46], even excluding four wild germplasm. We also found that the diversity level in this panel was much lower than that of rice [51,52] and wheat [32,53,54], which are also self-pollinating crops. That might be ascribed to the lower frequency of gene flow by introduction and utilization of external genetic accessions in Chinese sesame breeding programs [47]. Furthermore, 130 sesame lines could be classified into five types, including WIC(L), WLR, BIC, BLR and wild germplasm according to their sources. All subsets showed similar MAF, gene diversity, heterozygosity and PIC except for four wild germplasm collections. WIC(L) showed the lowest but quite wide variation of gene diversity and PIC than other subsets, which indicated a relatively narrow genetic basis in Chinese white seeded improved cultivars or inbred lines.

To get detailed knowledge of genetic relatedness among individuals (especially cultivars) in this panel, model-based STRUCTURE analyses were conducted and revealed the existence of two main groups in this sesame panel. The division of these two groups (G1 and G2) generally corresponds to their seed colors (white VS black) (Additional file 3: Table S3). Significant divergence between the two main groups was reflected by *F*_*st*_. Five subpopulations were identified within the 130 sesame accessions, which was cross-validated by STRUCTURE, PCA, NJ phylogenetic tree based analysis and AMOVA. Furthermore, most previous related studies in sesame revealed certain relationship between population structure and geographical distribution [24,46,47,49]. Our study of population structure revealed limited correlation with geographical distribution in P1, P2 and P4. Some earlier studies also indicated limited association between ecological or geographical origin and population differentiation in sesame [14,46]. Furthermore, 48 lines (36.9%) in this sesame panel were assigned into a mixed subgroup (Pmix) for low membership probability (< 0.60). Cho et al. [24] also categorized 27.3% of 150 sesame accessions as admixed forms with varying levels of membership shared among three genetic groups. 20.5% of 527 maize collection (a global germpasm) [55] and 35.5% of 155 maize inbred lines (mainly temperate germplasm) [48] were classified into a mixed group. This varied percentage of mixed lines may indicate various degree of gene flow by hybridization and introgression events.

### Impacts of selection and breeding on genetic diversity of Chinese sesame cultivars

Genetic diversity in sesame as in other crops has been reduced during domestication and breeding [56-58]. Nyongesa et al. (2013) also reported the genetic divergence between sesame and related wild species (2n = 32) in East Africa using ISSR markers. In this study, four wild germplasm accessions showed highest MAF, gene diversity, heterozygous and PIC. Population structure and differentiation analysis indicated that they (P5) were genetically far away from other sesame accessions in our panel. These wild germplasm accessions would therefore be useful in broadening genetic basis of traditional landraces and cultivars in China.

Furthermore, the genetic diversity and PIC of improved sesame cultivars was found to be lower than those of landraces, especially the white seeded cultivars. Greater differentiation of allele frequency was observed between BIC and BLR than WIC(L) and WLR lines. Compared to WLR or BLR lines, much more missing alleles than unique alleles were identified in WIC(L) or BIC lines, which indicates that the genetic basis was narrowed down during domestication and selection of mordent cultivars from landraces. Molecular genetic indices, such as MAF, gene diversity, PIC and allele frequency, all support that a declining genetic diversity occurred during the past five decades (from 1970s to 2010s) in China. Especially, compared to the Y2000s data, 7.7% missing alleles but only 1 unique allele were identified in the Y2010s.

In Chinese sesame breeding history, several important sesame cultivars had been developed and widely grown. The relationship among four families sharing common parents or progenitors, as well as among five subgroups suggested by STRUCTURE seems ambiguous, which might be caused by intercross of accessions belonging to different subgroups. In this study, several common alleles were identified in the four families, which can be used as important identification indexes of parentage or DNA fingerprinting for Chinese sesame cultivars. In addition, the common or unique alleles identified in different type, subgroups and families will be an important resource for marker-assisted breeding, in particular marker-assisted backcross or pyramiding breeding if more functional information are added by linkage or association mapping of important QTLs/genes. The five subgroups suggested by STRUCTURE in this study may provide breeders with more advices for broadening genetic basis of sesame cultivars toward better adaptability.

## Conclusions

This report presents the by far most comprehensive characterization of the molecular and genetic diversity of available sesame cultivars in China. We developed 349 SSRs and 79 InDel markers by a cDNA library and reduced-representation sequencing. Comparison of genetic diversity assessed by SSR and InDel markers confirmed that InDels are more polymorphic than SSRs but both showed comparable abilities for deciphering genetic diversity. Comparison of molecular marker information indicates that the genetic basis was narrowed down and the genetic diversity was declining during domestication and selection of mordent cultivars from landraces. Comparative analysis of allele distribution revealed genetic divergence between improved cultivars and landraces, even between cultivars released in different timelines. These results will be useful for assessing cultivars and for marker-assisted breeding in sesame.

## Methods

### Plant materials

Eighty two important Chinese improved sesame cultivars or lines (*Sesamum indicum* L., 2n = 26), including 70 white seeded improved cultivars or inbred lines [WIC(L)] and 12 black seeded improved cultivars (BIC) from major production areas, 44 landraces (*S. indicum* L., 2n = 26) representing geographically and phenotypically different sesame accession, and 4 wild germplasm accessions (putatively identified as *S. schinzianum*, *S. radiatum*, *S. malabaricum*, and *S. prostratum*) were used in this study (Additional file 3: Table S3). All of these lines had been self-pollinated for over five generations in Wuhan and Sanya to decrease the residual heterozygosity. Among these accessions, Zhongzhi 14 and Miaoqianzhima were chosen as a couple of templates for development of polymorphic markers and references for genotype determination, which show obviously different morphology in plant height, plant type, capsule shape, leaf shape and color, mature period, resistance and so on. All of these accessions were collected from the breeding units or the Sesame Middle Term Gene Bank at the Oil Crop Research Institute, Chinese Academy of Agricultural Sciences.

### Microsatellite marker development

In previous cooperative study, 1,949 non-redundant SSRs were identified from 1,688 unigenes in a cDNA library of Zhongzhi 14 [44]. Only SSR loci of perfect di-, tri-, tetra-, penta-, and hexanucleotide motifs with a minimum of 6, 4, 4, 4, and 4 repeats respectively were evaluated. Flanking oligonucleotide primers were designed using Primer 3 (http://bioinfo.ut.ee/primer3/), based on the following major parameters: PCR product size of 100-400 bp (optimal 200 bp), GC content of 40-70% (optimal 50%), annealing temperatures of 50-60°C (optimal 55°C), and primer length of 18–23 bases (optimal 20 bases).

Other published 466 EST-SSR markers and 134 genomic-SSR markers were also used in study (Table 1). The former included 342, 25, and 99 EST-SSR markers from HS [25,46], ZHY [33], and ZM [26,59] series respectively. The latter included 23 and 111 genomic-SSR markers of ‘GBssr’ [22,24] and ‘no.’ series (we named ‘GSSR’) [45], respectively.

### RAD sequencing and InDel marker development

We have combined the RAD approach with Illumina DNA sequencing for rapid and effective discovery of InDel markers for sesame. Genomic DNA of Zhongzhi 14 and Miaoqianzhima was extracted from leaves of three-week-old seedlings using the DNA extraction kit (TIANGEN Co. Ltd, Beijing), following the manufacturer’s instructions. The RAD library was constructed according to the protocol described by Baird et al. [60], restriction enzymes used were *EcoR* I and *Pst* I. Sequencing was carried out using the Illumina NGS platform HiSeq2000 at Major Biological Medicine Technology Co., Ltd. (Shanghai, China).

Solexa sequences at minimum coverage of 6X(about 2.4Gb each) were segregated by the barcode assigned to each sample. Reads of low quality (including reads with < 93 bp after trimming) or with ambiguous barcodes were discarded. After trimming each raw sequence read to 93 nucleotides from the 3’ end. For the RAD pair end based InDel calling, sequence reads from two materials were first grouped into clusters of identical sequences (RAD tags) and clusters using *Stacks*[61], with <7 or >200 sequences were discarded. Forward reads of two materials were grouped and the reads of other side (reverse reads) can also be grouped at this step. The reverse reads of each cluster of two materials were de novo assembled by *phrap* separately [62]. Then BLAST was used to compare the contigs generated by *phrap* from two materials. InDels (> 2 bp) were identified by gaps in alignment results, and regarded as true polymorphisms when each allele was observed at least three times.

### Genomic DNA extraction and PCR

Genomic DNA of 130 sesame accessions was all extracted from young leaves using the DNA extraction kit (TIANGEN Co. Ltd, Beijing). Polymerase chain reactions (PCR) for SSRs and InDels were performed in a 10 μl reactions, containing 10 ng DNA, 2 pmol of each primers, 2 nmol dNTPs, 15 nmol MgCl_2_, 0.2 U Taq DNA polymerase (Fermentas, Canada) and 1X PCR buffer supplied together with the enzyme. The PCR cycles were 94°C 3 min, 36 cycles of 94°C 20 s, 55°C ~ 60°C 30 s, 72°C 40 s, and a 5 min at 72°C for final extension. PCR products were separated in 8% non-denaturing polyacrylamide gels (Acr:Bis = 19:1 or 29:1) on a constant voltage of 180 V for 2 ~ 3 h, visualized by silver staining [63].

### Genotypic data analysis

For each polymorphic marker, the alleles present in each genotype were scored visually. The number of alleles, minor allele frequency, gene diversity, observed heterozygosity (H_e_), group-specific alleles, family shared alleles, polymorphic information content (PIC) and Nei’s genetic distance [64] were calculated using Powermarker version 3.25 [65,66]. Heterozygosity is simply the proportion of heterozygous individuals in the population. At a single locus it is estimated as $H=1-\sum_{i=0}^{k}x_{i}^{2}$. Gene diversity often referred to as expected heterozygosity, is defined as the probability that two randomly chosen alleles from the population are different. An unbiased estimator of gene diversity at the *l*th locus is ${\hat{D}}_{l}=\left(1-\sum_{u=1}^{k}{{\overset{\}}{p}}_{\mathrm{lu}}}^{2}\right)/\left(1-\frac{1+f}{n}\right)$. The polymorphism information content (PIC) is estimated as $\mathrm{PIC}=1-\sum_{i=1}^{k}p_{i}^{2}-\sum_{i=1}^{k-1}\sum_{j=i+1}^{k}2p_{i}^{2}p_{j}^{2}$. The significance of difference in gene diversity, PIC, allele frequency and other statistics was based on *P* value from Fisher’s exact test [67]. An analysis of *F*-statistics (*F*_*st*_) among populations was calculated using GENEPOP V4.2 [68]. The definition of *F*-statistics used here is $F_{\mathrm{ST}}\equiv \frac{Q_{2}-Q_{3}}{1-Q_{3}}$, Where the Q are probabilities of identity in state, Q2 among genes in different individuals within groups (populations), and Q3 among groups (populations).The model-based program STRUCTURE 2.34 [69,70] was used to infer population structure with SSRs and InDels. Five independent runs were performed setting the number of subpopulations (*k*) from 1 to 10, with 500,000 MCMC (Markov chain Monte Carlo) replications and a model for admixture model and correlated allele frequencies. The *k* value was determined by the log likelihood of the data (LnP(D)) in the STRUCTURE output and an ad hoc statistic ∆*k* based on the rate of change in LnP(D) between successive *k*[71]. Results of replicate runs from STRUCTURE were integrated by using the CLUMPP software [72]. Sesame accessions with membership probabilities ≥ 0.60 were assigned to the corresponding subgroup and accessions with membership probabilities ≤ 0.60 were assigned to a mixed subgroup [73]. In addition, principal component analysis (PCA) was conducted using the modules EIGEN implemented in NTSYS-pc 2.10 [74], and a neighbor-joining dendrogram was also constructed using the unweighted pair-group method (UPGMA) in NTSYS-pc 2.10. The hierarchical analysis of molecular variance (AMOVA) across all groups, subgroups and pairwise subgroups was performed using Arlequin V3.11 [75], with 1,000 permutations and sum of squared size differences as molecular distance.

## Abbreviations

AFLP: Amplified fragment length polymorphism; AMOVA: Analysis of molecular variance; BIC: Black seeded improved cultivars; BLR: Black seeded landraces; EST: Expressed sequence tag; Fst: *F*-statistics; He: Heterozygosity; InDel: Insertion-Deletion; ISSR: Inter-simple sequence repeat; MAF: Minor allele frequency; PCA: Principal component analysis; PIC: Polymorphic information content; RAD: Restriction-site associated DNA; RAPD: Random amplified polymorphic DNA; SRAP: Sequence-related amplified polymorphism; SSR: Simple sequence repeat; SNP: Single nucleotide polymorphism; UPGMA: Unweighted pair-group method; WIC[L]: White seeded improved cultivars or inbred lines; WLR: White seeded landraces.

## Competing interests

The authors declare that they have no competing interests.

## Authors’ contributions

K.W. and Y.Z.Z. designed research and wrote the manuscript; M.M.Y performed SSR markers analysis; H.Y.L. constructed this sesame panel and collected information of sesame cultivars; Y.T. performed RAD sequencing and InDel markers development; J.M. performed InDel markers analysis; K.W. performed EST-SSR markers development, analyzed data and result. All authors read and approved the final manuscript.

## Supplementary Material

Additional file 1: Table S1 Primer sequences, repeat types, repeat units, annealing temperature and expected PCR product sizes of 341 EST-SSRs identified from a cDNA library of Zhongzhi 14. Click here for file

Additional file 2: Table S2 Primer sequences, gap size, annealing temperature and expected PCR product sizes of 79 InDels identified from RAD sequencing of Zhongzhi 14 and Miaoqianzhima. Click here for file

Additional file 3: Table S3 Cultivar or accession, origin, type, releasing period, parentage and assignment of the genotypes assayed in this study. Click here for file

Additional file 4: Table S4 Allele frequencies of the whole sesame panel, different types, cultivars of different releasing period, and subgroups in this study. Click here for file
